# High-Dose Chemotherapy with Autologous Hematopoietic Stem-Cell Rescue for Pediatric Brain Tumor Patients: A Single Institution Experience from UCLA

**DOI:** 10.1155/2011/740673

**Published:** 2011-04-14

**Authors:** Eduard H. Panosyan, Alan K. Ikeda, Vivian Y. Chang, Dan R. Laks, Charles L. Reeb, La Vette Bowles, Joseph L. Lasky, Theodore B. Moore

**Affiliations:** UCLA Medical Center, Mattel Children's Hospital, Los Angeles, CA 90095, USA

## Abstract

*Background*. Dose-dependent response makes certain pediatric brain tumors appropriate targets for high-dose chemotherapy with autologous hematopoietic stem-cell rescue (HDCT-AHSCR). *Methods*. The clinical outcomes and toxicities were analyzed retrospectively for 18 consecutive patients ≤19 y/o treated with HDCT-AHSCR at UCLA (1999–2009). *Results*. Patients' median age was 2.3 years. Fourteen had primary and 4 recurrent tumors: 12 neural/embryonal (7 medulloblastomas, 4 primitive neuroectodermal tumors, and a pineoblastoma), 3 glial/mixed, and 3 germ cell tumors. Eight patients had initial gross-total and seven subtotal resections. HDCT mostly consisted of carboplatin and/or thiotepa ± etoposide (*n* = 16). Nine patients underwent a single AHSCR and nine ≥3 tandems. Three-year progression-free and overall survival probabilities were 60.5% ± 16 and 69.3% ± 11.5. Ten patients with pre-AHSCR complete remissions were alive/disease-free, whereas 5 of 8 with measurable disease were deceased (median followup: 2.3 yrs). Nine of 13 survivors avoided radiation. Single AHSCR regimens had greater toxicity than ≥3 AHSCR (*P* < .01). *Conclusion*. HDCT-AHSCR has a definitive, though limited role for selected pediatric brain tumors with poor prognosis and pretransplant complete/partial remissions.

## 1. Introduction

Certain pediatric brain tumors such as those of primitive neuroectodermal origin have demonstrated dose-dependent chemotherapy responses [1–3] and the principles of high-dose chemotherapy with stem-cell rescue have been applied to these pediatric brain tumors similarly to those of other solid tumors [4–6]. High-dose chemotherapy with autologous hematopoietic stem-cell rescue (HDCT-AHSCR) has been used successfully in children with recurrent/refractory or poor-prognosis medulloblastomas, primitive neuroectodermal tumors (PNETs), atypical teratoid/rhabdoid tumors, and central nervous system (CNS) germ cell tumors (GCTs) [7–17]. However, the efficacy of this treatment strategy for CNS tumors of glial origin, such as high-grade gliomas and ependymomas, is less encouraging [18–21]. The HDCT regimens reported in literature vary but mainly consist of alkylator-based myeloablation, including thiotepa (TT) with or without topoisomerase inhibitors [8, 13, 18–20, 22, 23]. Other agents may be rationally implemented in pre-HDCT chemotherapy depending on tumor type, such as vincristine (VCR) and methotrexate (MTX) for medulloblastomas or temozolomide (TMZ) for gliomas [1, 4, 6, 11, 12, 19, 21, 22, 24]. Variable intensity of regimens and numbers of myeloablations/AHSCRs may potentially result in different outcomes and toxicity profiles.

The disease histotypes, HDCT regimens, and patient characteristics have differed significantly among reported series [11, 12, 19, 22]. Nevertheless, achieving positive results generally is more likely in patients with no evidence of disease/minimal residual disease status at the time of stem-cell rescue [5, 23]. Because of this, with the exception of highly chemosensitive lesions like GCTs [13], the extent of surgical resection may play a crucial role in clinical outcomes. Surgery may also be used for second look pre- or posttransplant evaluations, since MRIs may reveal treatment-related heterogeneously enhancing lesions apparently indistinguishable from disease progression [6, 8, 25]. Thus additional long-term followup is imperative for more accurate outcome measurements. 

 HDCT-AHSCR is particularly attractive for pediatric brain tumor management, since it may permit omission of radiotherapy and its devastating neurocognitive sequelae, particularly in the very young [7, 8, 18]. It also can be used as a bridging modality for younger patients with radiosensitive lesions. Yet acute toxicity of high-dose chemotherapy itself may be unacceptable; while the main dose limiting hematopoietic suppression is reversed by AHSCR, excessive systemic toxicities including deaths have been reported [1, 20, 22–24]. In addition, modern advanced radiotherapy is less harmful [18, 26, 27], and its long-term consequences on the developing brain are presumably comparable to that caused by high-dose chemotherapy, which may not be negligible.

 HDCT-AHSCR for pediatric brain tumors has been regularly utilized at the University of California at Los Angeles (UCLA) since the late 1990s and we have conducted a retrospective review of consecutively treated patients to summarize our single institution's experience for this modality. While the limited statistical power and retrospective character of this study are limitations, it allows the derivation of valuable findings concurring with reported predictive variables and clinical outcome measurements. This is a reflective validation of a heterogeneous and small, yet representative patient group. It supports the results from evaluation of toxicity profile of various regimens utilized in a setting of standardized supportive care delivery. 

## 2. Methods

### 2.1. Patients

Informed consents were obtained from all 18 patients and/or parents in accordance with the UCLA Institutional Review Board (IRB# HSPC 10–000126). All 18 consecutive pediatric patients with brain tumors who underwent HDCT-AHSCR at UCLA from 1999 to 2009 were enrolled in this retrospective clinical review and outcome analyses (Table 1). The patients' data on demographic and clinical characteristics, treatments and disease status prior to AHSCR, complications after AHSCR, and final outcomes were extracted. For all patients, relevant clinical information including laboratory results was also retrieved by the hospital electronic medical record system. In addition to the electronic medical record, clinical information was supplemented by reviewing hard copies of patient medical records. 

### 2.2. Treatment

Once diagnosed with a brain tumor, accepted standard treatment was initiated for all patients based on tumor type and stage (Table 2). Indications for HDCT-AHSCR included young age prohibiting radiation therapy, recurrent/refractory or residual disease after upfront treatment, and/or chemosensitive primary tumors with poor prognoses. Patients with recurrent tumors were also treated with second-line chemotherapy with or without additional surgery and radiotherapy first, aiming for complete remission before the HDCT-AHSCR. Patients had at least a partial response or stable disease prior to HDCT-AHSCR. Peripheral blood stem-cells were harvested and then used in all cases as a source of hematopoietic stem-cells. Granulocyte colony stimulating factor was used for mobilization of peripheral blood stem-cells, which were collected, then stored unmanipulated in dimethyl sulfoxide and reinfused after HDCT.

Eight patients <3 years old who underwent 3 tandem AHSCR had myeloablative HDCT with carboplatin (CP), and thiotepa (TT), which were given once daily for 2 days on days −3 and −2 (Table 3). Seven of these 8 patients received 3 induction courses each with VCR, cisplatin (CDDP), cyclophosphamide (CPM) (with MESNA), and etoposide (VP). Six out of 9 patients with single AHSCR were conditioned utilizing CP on days −8, −7, −6; TT and VP on days −5, −4, −3. Two additional patients with nongerminomatous germ cell tumors (NGGCT) received a single AHSCR after receiving HDCT regimens consisting of TT and VP once daily on days −5, −4, and −3. Another patient with pineoblastoma received a single autologous transplant after HDCT with CPM for 4 days and melphalan for 3 days. Lastly, one patient with a supratentorial PNET who had 4 tandem AHSCRs received HDCT with CDDP, CPM, and VCR. Dose modification for CP was made for one patient who had abnormal renal function before the transplant.

Post-AHSCR, patients received standard supportive care measures, including bactrim and fluconazole for prophylaxis of pneumocystis jirovecii and opportunistic fungal infections, respectively. Transfusions of irradiated blood products were used to maintain hemoglobin level above 8 g/dl and platelet count above 20 × 10e3/*μ*L or higher for active bleeding for all patients. Granulocyte colony stimulating factor was utilized posttransplant if the patient had uncontrolled neutropenic sepsis or neutropenic fever unresponsive to antimicrobial agents. Supplemental parenteral nutrition was used to support patients with severe gastrointestinal mucositis with diminished caloric intake. Additional tumor-directed or palliative therapy was administered to three patients after transplant that had further residual, recurrent, or progressive tumors. Patient no. 9 with NGGCT received additional radiation therapy for palliation after recurrence post-AHSCR (Table 2), patient no. 5 started treatment with irinotecan, bevacizumab, and TMZ, and patient no. 8 was treated with irinotecan and TMZ for palliation. 

### 2.3. Definitions

Complete remission (CR) was defined as resolution of initially demonstrable tumors without the appearance of new diseased areas measured by MRI. In four cases, minimal residual heterogeneous post-treatment lesions enhancing on MRI were suspicious for refractory disease and were rebiopsied, which demonstrated absence of viable tumor and confirmed CR. Partial remission (PR) and stable disease (SD) were defined as greater or less than a 50% decrease in the product of the two largest perpendicular diameters of the tumor, respectively. If the initial cerebrospinal fluid (CSF) cytology was positive, two consecutively negative CSF cytology exams were required to document CR or PR. Repeatedly positive CSF without persistent increase in cell count was an additional finding that qualified for stable disease. Progressive disease was defined as an increase of >25% in tumor area with maximum perpendicular diameters in any site of residual disease area compared to immediate pretreatment area or compared to area of best prior response at that site, the appearance of a new demonstrable lesion, or the conversion of CSF cytology from negative to positive. Neutrophil engraftment was defined as an absolute neutrophil count recovery from nadir to 0.5 × 10e3/*μ*L or greater for 2 consecutive days. Toxicity grading was conducted based on the standard National Cancer Institute toxicity criteria. Progression-free survival was assessed from the date of the first AHSCR to the date of disease relapse or progression. Overall survival was assessed from the date of first AHSCR to the date of death. 

### 2.4. Statistics

Both progression-free survival (PFS) and overall survival (OS) were related to the explanatory variables using a Cox proportional hazards model and illustrated with the Kaplan-Meier method as implemented by Stata 8.0 (StataCorp). Multivariate logistic regression models were performed to control for extent of resection and disease status prior to AHSCR. Analysis of variance (ANOVA) was used to test the significance of toxicity differences between single versus ≥3 AHSCR regimens. This was further confirmed by the nonparametric K-Wallis test. All *P* values were two sided and *P* < .05 was considered significant. 

## 3. Results

### 3.1. Patient Characteristics

A total of 18 patients were identified in our comprehensive Pediatric Blood and Marrow Transplant database, who had HDCT-AHSCR from 1999–2009 for brain tumor treatment. Patient characteristics are presented in Table 1. Patients were 0.4–19 years old at diagnosis (mean 5.6, median 2.3 yrs), and male: female ratio was 2 : 1. Two thirds of the patients had neuronal/embryonal tumors (7 medulloblastomas, 4 PNETs, and a pineoblastoma), two patients had anaplastic glial tumors (an ependymoma and an oligodendroglioma), one patient had a neurocytoma, and three had NGGCT. Patients with NGGCT were >15 y/o, and the majority of the remaining patients were younger (<5–3 y/o). Locations of these lesions are shown in Table 1. Only four patients had recurrent lesions; the rest had primary tumors. Chang staging principles applied to all tumors and revealed 4 patients with M2, four with M3 (including positive CSF cytology and focal, subarachnoid, or leptomeningeal spreads) and the remaining with M0 status as a highest stage at any time prior to HDCT-AHSCR. 

### 3.2. Treatment

Most of the patients had initial surgical intervention: eight ended up with gross total resection (GTR) on first attempt, 7 had subtotal resections (STR), 2 of which were followed by GTR, and 3 NGGCT were not resected initially (one eventually had GTR after recurrence) (Table 2). A total of 11 patients had *ventriculo*-peritoneal shunt placement.

Various agents and combinations were utilized for initial chemotherapy as shown in Table 2. With the exception of one patient with pineoblastoma, who was treated with initial radiotherapy and subsequent temozolomide only after recurrence (prior to HDCT), the rest received chemotherapy upfront. This patient with pineoblastoma was conditioned with CPM and Melphalan for a single AHSCR. Three NGGCT patients received initial VP, ifosfamide, and CP × 6 courses, one of whom also got radiotherapy prior to recurrence. These patients with NGGCT were conditioned with VP/TT (+CP for patient no. 9 with recurrent NGGCT) for single AHSCR. One older patient (no. 1) with medulloblastoma was initially treated at an outside hospital with a combination of CDDP, VCR, Lomustine, and radiotherapy prior to recurrence. The patient with recurrent neurocytoma received initial chemotherapy with TMZ and CPM. 

The remaining 12 patients were initially treated with at least three courses of VCR, CDDP, CPM, and VP; 4 of whom had 5 courses with addition of MTX, and 2 of these 4 patients also received TMZ. Seven out of these 12 patients (as well as patient no. 8 with recurrent neurocytoma) received 3 cycles of high-dose CP and TT, each followed by AHSCR, and one of the 12 received a different HDCT regimen and ×  4 AHSCRs as described in the methods section and shown in Table 3. Four patients who had additional TMZ and/or MTX to initial chemotherapy and patient no.1 also received VP, in addition to CP and TT (Table 3) during HDCT for AHSCR. Four patients with recurrent disease also received additional salvage therapy prior to AHSCR as shown in Table 2. For one of them, HDCT with TT and CP for AHSCR served also as a salvage regimen for residual/SD medulloblastoma. 

In summary, half of our patients received ≥3 tandem AHSCR with generally less prior chemotherapy exposure during induction, and milder HDCT per each AHSCR. The other half got stronger induction chemotherapy and more intensive HDCT prior to their single AHSCR. This dichotomization among the patients allowed us to evaluate for differences of toxicity profiles and test the hypothesis that toxicity profiles of single AHSCR versus ≥3 tandem AHSCR regimens are different.

The median diagnosis to transplant time was 6 months (mean: 1 year). The average number of infused CD34^+^cells per kg per AHSCR was 20 × 10e6 (range: 2 to 100 × 10e6). The average time between the tandem courses was 29 ± 7 days (Mean ± SDEV, range 21–53) (this is versus intended 21 days, mostly due to delayed hematopoietic engraftment/count recovery). Neutrophil engraftment was estimated by an average of 17 ± 8 (SDEV) days post-transplant. Ten patients did not receive any radiotherapy during their entire treatment at the time of our analyses. Three patients (no. 1, 10, and 13) received cerebrospinal radiotherapy prior to AHSCR, four patients (no. 2, 4, 15, and 18) received cerebrospinal radiotherapy post-AHSCR, and one patient (no. 9) received radiotherapy both pre- and post-AHSCR. 

### 3.3. Outcome Analyses

**Figure 1:**
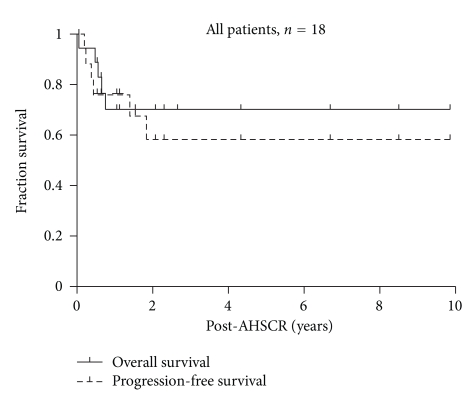
Kaplan-Meier survival estimates for PFS and OS, all patients (*n* = 18).

The follow-up durations were 3 ± 2.4 years (mean ± SDEV, range 0.9–8.9, and median: 2 years) after diagnosis and 2 ± 2.4 years after transplant (range 0.05–8.5, and median: 1.1 years). The probabilities of three-year PFS and OS from AHSCR were 60.5% ± 16 (mean ± Std. Error) and 69.3%  ± 11.5, respectively, (Figure 1). 

We sought out associations between the clinical outcome and the following parameters to identify potential prognostic predictors: age, gender, tumor type and staging, extent of surgical resection, chemotherapy regimens, radiation therapy, disease status prior to AHSCR, as well as grade of toxicity as another measurable endpoint. Heterogeneous patient characteristics and tumor histotypes along with small numbers were restrictive for appropriate statistical power for some relevant analyses, such as testing different regimens within tumor subtypes. Nevertheless, in univariate analyses we found no general associations between the age, gender, tumor type/staging, HDCT regimens (single versus ≥3 AHSCR), radiation therapy, toxicity grade, and clinical outcome expressed either as PFS and OS for all 18 patients. 

**Figure 2:**
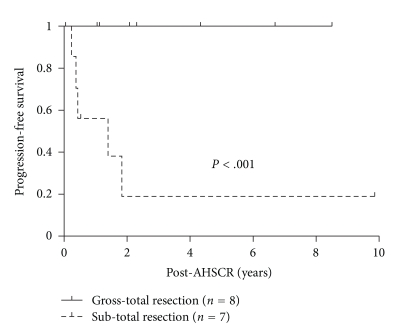
Kaplan-Meier survival estimate for PFS for patients with initial subtotal resection (STR) (*n* = 7) versus those with initial gross total resection (GTR) (*n* = 8). Three patients with nongerminomatous germ cell tumors did not have initial surgery and are not included in this analysis.

**Figure 3:**
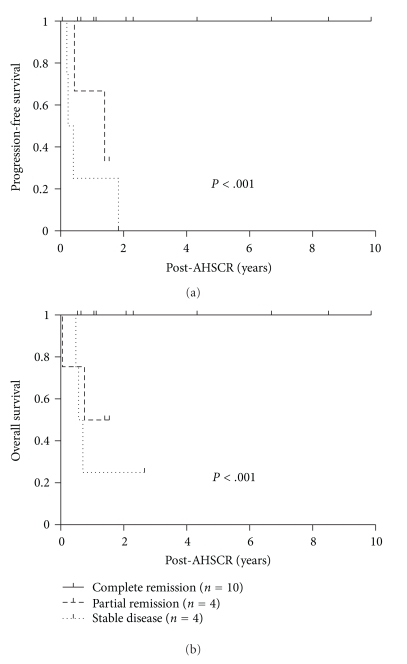
Kaplan-Meier survival estimate for PFS (a) and OS (b) according to disease status prior to AHSCR.

Our significant findings included a strong correlation between the extent of initial surgical resection and PFS (Figure 2), as well as the disease status prior to AHSCR and clinical outcome (both PFS and OS, as shown in Figures 3(a) and 3(b), respectively). Figure 2 demonstrates the Kaplan-Meier curves for PFS of 15 patients with initial resection (excluded are 3 NGGCT patients), separated according to GTR versus STR. Patients with initial STR have significantly worse PFS than those with GTR (*P* < .001, Hazard Ratio (HR) = 9, and 95% Confidence Interval (CI) >10−>10 per Cox proportional Hazards regression).

 Figures 3(a) and 3(b), respectively, depict Kaplan-Meier curves for PFS and OS for all 18 patients, based on their disease status prior to AHSCR. Patients with CR prior to AHSCR (either biopsy proven negative or not excluded minimal residual disease) have sustained >3 yr PFS and OS of 100%, whereas most patients with PR or SD have succumbed due to disease progression (one patient died from therapy-related toxicity). Cox proportional Hazards regression demonstrated *P* < .001, HR = 6.52, with 95% CI = 2.67–15.9 for PFS, and *P* < .001, HR = 4.98, with 95% CI = 1.86–13.4, for OS. 

Multivariate analysis was performed to test interdependence between the extent of initial surgical resection and disease status prior to AHSCR as predictors of PFS. This demonstrated, that when adjusted for each other, GTR independently sustains its predictive significance with *P* < .001, HR > 10, CI> 10−> 10, with CR prior to AHSCR approaches being independently significant (*P* = .056, HR = 2.8, CI = 0.97–7.9). In concordance, 87.5% of patients with GTR had CR prior to AHSCR and 70% of CR patients had GTR on initial resection. 

### 3.4. Toxicity

**Figure 4:**
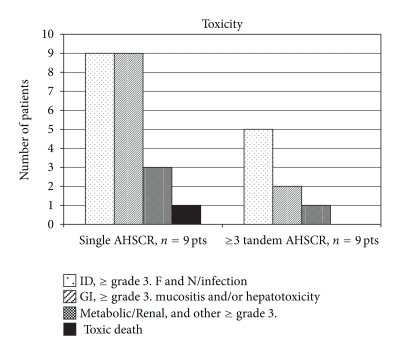
Summative toxicity of regimens with single AHSCR and ≥3 tandem AHSCRs. ID: infectious diseases, F&N: Fever and neutropenia, GI: gastrointestinal.

Table 4 demonstrates detailed toxicity data for all 18 patients as graded by National Cancer Institute criteria. This data comprehensively captures toxic episodes for all listed categories within 30 days after AHSCR. In addition, all patients experienced grade 3-4 hematopoietic toxicities as anticipated requiring irradiated blood product support. As summarized in Figure 4, patients with ≥3 AHSCR experienced less toxicity in general, whereas patients who received single AHSCR had more toxic episodes and 1 toxic death.

To quantify and compare toxicity data, we conditionally scored all patients' toxicity other than hematopoietic suppression; those who had no other grade 3 or higher toxicity were scored with 0 (*n* = 4), fever and neutropenia, infectious complications were scored with 1 (*n* = 3); and fever and neutropenia with at least one more organ system grade 3 or higher toxicity (excluding hematopoietic) were scored with 2 (*n* = 11). These scores were first used as variables to perform ANOVA between single AHSCR and ≥3 AHSCR groups, and it confirmed the significant difference (*P* < .001, at ≥3 AHSCR mean toxicity score (±SDEV) was 0.78 ± 0.83, and at single AHSCR mean toxicity score was 2 ± 0). Furthermore, these results were confirmed by performing the nonparametric Kruskal-Wallis test between two groups (*P* = .0054).

There was no formal neuropsychological testing for most patients; however, Lansky-Karnofsky performance scores demonstrated that all survivors had satisfactory functional status (Table 3). 

## 4. Discussion

This retrospective study demonstrates that pediatric brain tumor patients treated with HDCT-AHSCR are most likely to have favorable outcome if patients achieved CR (either biopsy proven or by radiologic criteria, that is, minimal residual disease not excluded) at the time of AHSCR. Gross total resection, which has historically been one of the most prominent favorable prognosticators, [22, 28], was associated with CR in our study and independently predicted PFS. In contrast, patients with SD at the time of transplant and especially those who had progression prior to transplant had the poorest chance of survival when treated by HDCT-AHSCT. Our data also suggests that some of the patients with PR without overt progression prior to transplant still may be salvaged by HDCT-AHSCT. These findings are in concordance with multiple previous reports [8, 22, 23]. Our 60% PFS and 69% OS rates are also comparable or superior with reported outcome data [6, 7, 12, 18, 19, 21, 22]. These survival rates rationalize the use of this modality for pediatric brain tumor patients with poor prognosis, with estimated EFS/OS generally less or equal than 50–40% at best, if treated with nonmyeloablative therapies [1, 5, 23]. In our study, it is not possible to conclude whether a portion of the included patients already were cured without the HDCT-AHSCR. International consensus meetings have concluded that the precise role of HDCT-AHSCR in pediatric brain tumors can only be determined in randomized trials.

 The heterogeneity of tumor types and treatment regimens prevented the development of any strong conclusions based on the negative findings of our retrospectively studied small patient group. Particularly, subgrouping patients into embryonal tumors (compared with others) and/or further subdivision according to treatment regimens result in small numbers, which cannot be utilized for conclusive analyses. Nevertheless, only 25% (3/12) of patients with embryonal tumors experienced recurrence after AHSCR, compared to 60% (2/3) of patients with glial/mixed lesions (Table 3), an observation that concurs with well-established knowledge that medulloblastomas and PNETs have better responsiveness [7, 8, 11, 12, 14, 19, 22, 24] to this modality compared to glial tumors [6, 11, 18–20, 22]. In addition, we cannot exclude that more intensive induction with stronger single HDCT/AHSCR harbors some therapeutic advantage over less intensive induction and “milder” HDCT with ≥3 AHSCR for embryonal tumors, since 2 out of 3 recurrences in 12 embryonal tumors occurred among the 6 patients, who got ≥3 AHSCR. Yet, our one young patient with anaplastic ependymoma is in sustained CR 4.3 yrs post-triple tandem AHSCR regimen (no radiation), despite the literature which reports very poor outcome (5-yaer EFS 12%) for those treated with single AHSCR [20].

While the aforementioned comparisons of clinical outcome between single and ≥3 AHSCR regimens in subgroups lack significant power, our toxicity data analyses are more robust (assuming that tumor type is unrelated to toxicity). There are no randomized studies between regimens with single versus ≥3 AHSCR reported in the literature. However, up to 10–20% toxic mortality has been documented in series with stronger thiotepa-based regimens and single-AHSCR [19, 20, 22–24], whereas a larger medulloblastoma study with milder cyclophosphamide-based chemotherapy and up to 4 AHSCRs had no protocol-related deaths [12]. Our data support these findings in that our only toxic death occurred in the single AHSCR group (1/9, 11%). In addition, regimens with ≥3 AHSCR were tolerated much better with significantly fewer toxic episodes (Table 4, Figure 4). It appears that repetitive administration of TT at doses comparable to that used in single transplant (which utilizes concomitant VP in HDCT) may decrease nonhematologic toxicity and allow administration of a greater cumulative drug dose. Whether this reduced toxicity is accompanied with decreased efficacy still remains to be seen. 

The major papers on transplant in embryonal brain tumors in children are reported on the Head Start program byFangusaro et al., which is referenced above [8]. The other approach to embryonal recurrent tumors is enhanced delivery by intra-arterial chemotherapy and osmotic blood-brain barrier disruption (IA/BBBD), as recently reviewed in Cancer by Jahnke et al. [29]. Comparison of these two larger series with regards of efficacy demonstrates comparable disease-free and OS survival rates of around 40% and 50%, respectively, at about the 3-year mark. Our patients' outcome with an almost 60% PFS and 70% OS at 3 years may seem promising; however, many of the common critical objections apply. First, only 2 of our 12 patients with embryonal tumors had recurrent tumors; the remaining had primary tumors (Table 1). Second, our inclusion criterion for this study is the high-dose therapy/autologous rescue and not the intention to treat. Moreover, our study is nonrandomized and followup is rather short. Nevertheless, our survival curves apparently are reaching their plateau, and we anticipate ≥50% long-term survivorship. In general, small, heterogeneous, and selected study groups hamper studies in this field. According to newer data, medulloblastomas with particular histological features and molecular signatures may have a more favorable prognosis [30], but the retrospective nature of our study does not allow us to perform these analyses.

Toxicities observed in our patients were similar to pediatric supratentorial PNET patients reported by Fangusaro et al. [8] and for children with medulloblastoma [31]. These mainly involved grade 3-4 hematopoietic toxicities, episodes of febrile neutropenia and, mucositis, often requiring narcotics. Transient liver transaminase elevations were also observed during chemotherapy. In contrast, in IA/BBBD study reported hematopoietic suppression and neutropenic fever to a lesser extent [29], highlighting the advantage of less systemic toxicity by this modality.  Table 5 summarizes selected nonneurological toxicities of these three studies in general. In addition, regarding neurological toxicities, IA/BBBD was reportedly associated with neurotoxic episodes in approximately 30% of patients with a vast majority with reversible neurologic deficit lasting >24 to 48 hrs. This is comparable to the neurotoxicity observed for our 18 patients, a third of whom have experienced neurological adverse effects in one form or another, including grades 1 and 3 hearing loss (Table 4). In comparison, approximately 60% of patients sustained high-frequency hearing loss necessitating a reduction or elimination of cisplatin in study reported by Fangusaro et al. [8]. Patients in the IA/BBBD study received heterogeneous sodium thiosulfate regimens, and an accurate comparison of ototoxicity was not feasible [29]. 

A number of mechanisms by which dose-intensive chemotherapy might help include steep dose response curve of alkylating agents [1–3, 32], blood-brain-barrier penetration [33, 34], and overcoming intrinsic resistance of hypothetical subpopulation(s) of cells such as brain tumor initiating cells [35, 36]. While HDCT-AHSCR may offer these advantages, we conclude that further intensification of regimens using HDCT likely has reached the point of maximum clinical toxicity, while resulting in diminished return, necessitating the development of newer modalities to improve upon the efficacy of brain tumor therapy. Recent advances in the molecular pathology of malignant glioma and medulloblastoma provide more opportunities for targeting brain tumors [30, 37]. This knowledge might be used for proposed biological modifications following HDCT-AHSCR for pediatric brain tumors [38], and pediatric brain tumor derived neurospheres may also provide an excellent predictive model for preclinical explorations for such important endeavors [39]. 

## Figures and Tables

**Table 1:** Patient characteristics.

Patient number	Age at diagnosis (years)/Gender	Diagnosis, grade	Location	Primary/recurrent	Highest stage
1	10.9/Male	Medulloblastoma, IV	Posterior fossa and Cervical-spine	Recurrent	M3
2	15.9/Male	NGGCT	Pineal	Primary	M0
3	0.9/Male	Medulloblastoma, IV	Posterior fossa	Primary	M0
4	17.3/Male	NGGCT	Pineal	Primary	M0
5	2.9/Female	Anaplastic Oligodendroglioma, III	Frontal, Left	Primary	M0
6	5.3/Male	Supratentorial PNET, IV	Cerebral, Left	Primary	M0
7	0.6/Male	Medulloblastoma, IV	Posterior fossa	Primary	M2
8	1.2/Female	Neurocytoma, II	Frontal, Right	Recurrent	M3
9	18.6/Male	NGGCT	Pineal	Recurrent	M3
10	4.4/Female	Supratentorial PNET, IV	Frontal, Left	Primary	M0
11	1.8/Male	Supratentorial PNET, IV	Frontal, Right	Primary	M0
12	2.2/Male	Anaplastic Ependymoma, III	Occipitoparietal, Left	Primary	M0
13	10.2/Female	Pineoblastoma, IV	Pineal	Recurrent	M2
14	0.4/Male	Medulloblastoma, IV	Posterior fossa	Primary	M3
15	2.4/Female	Medulloblastoma, IV	Brainstem	Primary	M0
16	2.1 /Female	Medulloblastoma, IV	Posterior fossa	Primary	M0
17	2.1/Male	Supratentorial PNET, IV	Frontal, Right	Primary	M2
18	1.7/Male	Medulloblastoma, IV	Posterior fossa	Primary	M2

NGGCT: nongerminomatous germ cell tumor.

PNET: primitive neuroectodermal tumor.

**Table 2:** Surgeries, chemotherapy prior to HDCT-AHSCR, and radiotherapy.

Patient number	Initial surgery	Initial chemotherapy	Salvage chemotherapy	Radiotherapy (timing)
1	GTR	CDDP, VCR, Lomustine	CPM, Topotecan	CSI (pre-recurrence/AHSCR)
2	—	CP, VP, Ifos	none	CSI (post-AHSCR)
3	STR	CDDP, VCR, CPM, VP, MTX	none	None
4	—	CP, VP, Ifos	none	CSI (post-AHSCR)
5	STR	CDDP, VCR, CPM, VP	none	None
6	GTR	CDDP, VCR, CPM, VP, MTX, TMZ	none	None
7	GTR	CDDP, VCR, CPM, VP, MTX, TMZ	none	None
8	STR	TMZ, CPM	ICE, CPM, Topotecan	None
9	—	CP, VP, Ifos	ICE	CSI (pre-recurrence and post-AHSCR)
10	GTR	CDDP, VCR, CPM, VP	none	CSI (pre-AHSCR)
11	GTR	CDDP, VCR, CPM, VP, MTX	none	None
12	GTR	CDDP, VCR, CPM, VP	none	None
13	STR	None	TMZ	CSI (pre-recurrence/AHSCR)
14	STR	CDDP, VCR, CPM, VP	CP, Thiotepa (HDCT)	None
15	STR	CDDP, VCR, CPM, VP	none	CSI (post-AHSCR/recurrence)
16	GTR	CDDP, VCR, CPM, VP	none	None
17	GTR	CDDP, VCR, CPM, VP	none	None
18	STR	CDDP, VCR, CPM, VP	none	CSI (post-AHSCR)

GTR: gross total resection, STR: subtotal resection, CDDP: Cisplatin, VCR: Vincristine, CP: Carboplatin, VP: Etoposide, Ifos: Ifosfamide, CPM: Cyclophosphamide, MTX: Methotrexate, TMZ: Temozolomide, CSI: Cerebrospinal irradiation, ICE: Ifos, CP, and VP.

**Table 3:** HDCT-AHSCR and outcome.

Patient number	Age at first AHSCR (years)	Status at AHSCR	HDCT prior to AHSCR	Number of AHSCR	Recurrence after transplant	Outcome	Latest Lansky/Karnovsky performance scores
1	15.2	PR	^¶^CP, TT, VP	1	No	Deceased	—
2	16.4	CR	$\hat{\;}$VP, TT	1	No	Alive	100
3	1.6	CR	^¶^CP, TT, VP	1	No	Alive	90
4	17.8	PR	$\hat{\;}$VP, TT	1	No	Alive	90
5	3.2	PR	*CP, TT	3	Yes	Alive	90 (Prior to recurrence)
6	5.9	CR	^¶^CP, TT, VP	1	No	Alive	90
7	1.2	CR	^¶^CP, TT, VP	1	No	Alive	90
8	2.8	SD	*CP, TT	3	Yes	Alive	90
9	20.0	SD	^¶^CP, TT, VP	1	Yes	Deceased	—
10	4.9	CR	CDDP,VCR,CPM	4	No	Alive	100
11	2.3	CR	^¶^CP, TT, VP	1	No	Alive	90
12	2.5	CR	*CP, TT	3	No	Alive	100
13	15.3	SD	CPM, Melphalan	1	Yes	Deceased	—
14	0.7	SD	*CP, TT	3	Yes	Deceased	—
15	2.7	PR	*CP, TT	3	Yes	Deceased	—
16	2.4	CR	*CP, TT	3	No	Alive	100
17	2.5	CR	*CP, TT	3	No	Alive	100
18	2.0	CR	*CP, TT	3	No	Alive	90

PR: Partial remission, CR: Complete remission, SD: Stable disease. ^¶^CP, TT, VP: Carboplatin (500 mg/m^2^/dose or 16.7 mg/kg/dose for <3 y/o) on days −8, −7, and−6; Thiotepa (300 mg/m^2^/dose or 10 mg/kg/dose for < 3 y/o) and Etoposide (250 mg/m^2^/dose or 8.3 mg/kg/dose for <3 y/o) on days −5, −4, and−3, *CP, TT: Carboplatin 17 mg/kg/dose, and Thiotepa 10 mg/kg/dose, given IV over 2 hours once daily for 2 days on days −3 and −2, $\hat{\;}$VP, TT: Thiotepa 300 mg/m2/dose and Etopposide 500 mg/m2/dose once daily on days −5, −4, and −3, CDDP: Cisplatin, CPM: Cyclophosphamide, and VCR: Vincristine.

**Table 4:** Toxicity.

Patient number	Infectious complications	GI mucositis	Liver, transaminitis (hyperbilirubinemia)	Metabolic/renal	Other
1	Grade 4, F&N	Grade 3	Grade 3 (Grade 3)	Grade 4 (elevated BUN/Creatinine)	Neuro: Grade 3, Altered mental status Cardiovascular: Grade 4, Hypotension Pulmonary: Grade 4, Respiratory failure Skin: Grade 4, TEN
2	Grade 3, F&N	Grade 3			
3	Grade 3, F&N	Grade 3	Grade 3		
4	Grade 3, F&N, Herpetic stomatitis	Grade 3			Endo: Exacerbation of baseline panhypopituitarism
5	Grade 3, F&N				
6	Grade 3, F&N, Serratia UTI	Grade 3		Grade 4, Hypokalemia	CV: Grade 1, Prolonged QTC Neuro: Grade 3, Hearing loss
7	Grade 3, Fever, sepsis with Pseudomonas and Klebsiella	Grade 3			
8	Grade 3, F&N, UTI with Vancomycin-resistant enterococcus	Grade 3	Grade 2		Neuro: Grade 2, Self-resolved seizure
9	Grade 3, F&N	Grade 3			
10				Grade 1, Decreased GFR	Neuro: Grade 1, High-frequency hearing loss
11	Grade 3, F&N, Eenterococcus UTI	Grade 3			
12				Grade 1, Hypomagnesemia	Neuro: Grade 1, High frequency hearing loss
13	Grade 3, Pneumonia	Grade 3		Grade 1, Hypokalemia Grade 3, Hyponatremia (not SIADH)	
14	Grade 2, Fever Grade 3, F&N		Grade 2 (Grade 3)		Neuro: Grade 4, Status epilepticus
15				Grade 1, Hypomagnesemia	
16					
17	Grade 3, F&N			Grade 1, Hypomagnesemia	
18	Grade 3, Zoster infection				

F&N: Fever and neutropenia, UTI: Urinary tract infection, GFR: Glomerular filtration rate, SIADH: Syndrome of inappropriate antidiuretic hormone hypersecretion, TEN: Toxic epidermal necrolysis.

**Table 5:** Comparison of nonneurological toxicity of three studies.

Study	IA/BBBD for embryonal/germ cell tumors/Jahnke et al. [29]	High-dose chemotherapy and autologous transplantation for sPNETs/Fangusaro et al. [8]	Current series
Number of patients	54	43	18
Hematopoietic	~35%	100%	100%
Fever and neutropenia	24%	70%	60%
Mucositis	—	Frequent in patients receiving methotrexate	56%
Toxic mortality	— (No IA/BBBD treatment-related deaths were reported; all 3 patients who died from delayed neurotoxicity received CSI).	5% (infection associated)	6% (multiorgan failure)

IA/BBBD: Intra-arterial Chemotherapy and Osmotic Blood-Brain Barrier Disruption.

sPNETs: supratentorial Primitive Neuroectodermal Tumors.

CSI: Cerebrospinal Irradiation.
